# Estimating Postural Stability Using Improved Permutation Entropy via TUG Accelerometer Data for Community-Dwelling Elderly People

**DOI:** 10.3390/e22101097

**Published:** 2020-09-29

**Authors:** Chia-Hsuan Lee, Shih-Hai Chen, Bernard C. Jiang, Tien-Lung Sun

**Affiliations:** 1 Department of Industrial Management, National Taiwan University of Science and Technology, Taipei 106, Taiwan; sweat0430@mail.ntust.edu.tw (C.-H.L.); bcjiang@mail.ntust.edu.tw (B.C.J.); 2 Department of Industrial Engineering and Management, Yuan Ze University, Taoyuan 320, Taiwan; s1038905@mail.yzu.edu.tw

**Keywords:** postural stability, timed up and go, inertial sensor, permutation entropy, weighted-permutation entropy, community-dwelling elderly

## Abstract

To develop an effective fall prevention program, clinicians must first identify the elderly people at risk of falling and then take the most appropriate interventions to reduce or eliminate preventable falls. Employing feature selection to establish effective decision making can thus assist in the identification of a patient’s fall risk from limited data. This work therefore aims to supplement professional timed up and go assessment methods using sensor technology, entropy analysis, and statistical analysis. The results showed the different approach of applying logistic regression analysis to the inertial data on a fall-risk scale to allow medical practitioners to predict for high-risk patients. Logistic regression was also used to automatically select feature values and clinical judgment methods to explore the differences in decision making. We also calculate the area under the receiver-operating characteristic curve (AUC). Results indicated that permutation entropy and statistical features provided the best AUC values (all above 0.9), and false positives were avoided. Additionally, the weighted-permutation entropy/statistical features test has a relatively good agreement rate with the short-form Berg balance scale when classifying patients as being at risk. Therefore, the proposed methodology can provide decision-makers with a more accurate way to classify fall risk in elderly people.

## 1. Introduction

In the information age, it is no longer difficult to obtain data; rather, the difficulty lies in extracting only the relevant, important, and non-redundant information [1]. When elderly people fall, they can incur serious health problems that can cause physical and psychological trauma, which can increase stress on the health care system. About one-third of elderly people fall every year, and the chance of falling increases with age [2,3]. Falling can have serious long-term consequences for elderly people, including hospital injuries, decreased mobility, fear of falling, and even death. Older people with gait or balance problems are at higher risk of falling in the future [4,5]. To develop an effective fall prevention program, elderly people at risk of falling must first be identified before appropriate interventions can be implemented to reduce or eliminate of preventable falls. Researchers have indicated that more than 50% of potential falls associated with older people have been avoided due to ongoing fall prevention interventions [6]. Researchers have also performed extensive motion evaluation in recent years, mostly using questionnaires [7,8,9,10] or traditional indictor methods for analysis, such as time, step count, and even exploration techniques for extracting overlooked information in databases [9,11,12], which are gaining popularity in the medical field. However, Sun and Sosnoff [13] found that the variation in measured parameters, assessment tools, sensor sites, movement tasks, and modeling techniques precludes a firm conclusion on their ability to predict future falls. The timed up and go (TUG) test is a widely used and accepted clinical test for assessing basic functional mobility [14]. It is simple and easy to administer anywhere and anytime. Zakaria et al. (2015) [15] attempted to use inertial sensors in two risk groups categorized into low fall and high fall risk with 13.5 s duration taken to complete the TUG test as the threshold between them, followed by analysis being carried out in phases. However, they found that the fall-risk evaluation using the time duration parameter could not interpret the activity in each phase; therefore, they suggested using relevant parameters related to appropriate signals and directions to reveal the activities of a subject in each phase. Furthermore, Lee et al. (2016) [16] attempted to use entropy to solve the problem based on multiscale entropy (MSE) indicators. Specifically, they found that all are distinguishable in TUG, the Berg balance scale (BBS), and the combined TUG and the short-form Berg balance scale (SFBBS), indicating that MSE can effectively classify the participants of these clinical tests using behavioral actions. Jian et al. (2020) [17] also employed entropy with video analysis and machine learning to predict the TUG score from gait characteristics. Their results showed that copula entropy is used to select the characteristics that are mainly related to the TUG score. The selected characteristics were then fed into the predictive models to predict the TUG score whereas experiments on the data demonstrated the effectiveness of the proposed method. However, overall, considerable future work is needed to determine a clinical, meaningful, and easy-to-interpret fall-risk diagnosis utilizing sensing technology. Feature selection, used to guide the selection of meaningful features to establish effective decision-making models, may allow for improved early diagnosis using limited data.

Although continuously monitoring patients’ gait and balance may reduce preventable falls through early warnings and appropriate interventions [18], this requires extensive professional resources (e.g., physiotherapists, nurses, and doctors). Wearable systems based on inertial sensors that are light, portable, and inexpensive allow body motion to be quantitatively measured and thus can fill the gap between patient needs and the available resources. Feature calculation of sensor data has been based mainly on statistical descriptions regarding the gait, time, and other features that can be calculated without an inertial sensor [19,20,21]; however, the availability and significance of the representative of the acceleration and rotation angle collected by the sensor itself are less relevant areas of research [15].

Quantifying the complexity of a given physiological time series is an important challenge in data analysis that can provide deeper insight into the potential mechanisms of physiological signals. For this, entropy measuring, such as Shannon entropy [22], approximate entropy [23], or sample entropy [24], is commonly employed. However, the classical Shannon entropy [22] ignores the temporal order of the values in a time series, whereas the sample entropy requires high complexity calculations. One of the key points of decision making is immediate response. MSE has been employed as a feature to explore its availability [16,25]. However, although a good classifier, MSE is difficult to use in making real-time decisions. In response, Bandt and Pompe [26] combined the concepts of entropy and symbolic dynamics to measure the complexity via permutation entropy (PE) in 2002. PE is computationally simple, efficient, and robust, and thus is preferable when using large data sets [27]. Furthermore, data analysis employing PE can be reduced to a comparison of values in different time series to compare the complexity of the underlying processes [26], such as physical medicine and rehabilitation. Although PE has been used to analyze gait data, e.g., to classify normal and pathological gait, only analyzing the sample order without considering the amplitude may adversely affect the performance of PE. Riedl et al. [28] also found that as the values of PE depend on the chosen parameters, the presence of multiple parameter pairs can cause a multiple testing problem. Other methods have thus been proposed to address this possible weakness of PE, such as weighted-permutation entropy (WPE) [29]. WPE was devised to account for the variability of amplitudes that result in the same motif [29]; generally, we attempt to use PE and WPE to add the availability to determine the features are most indicative of fall risk. Moreover, because of the multiple factors of falls, postural stability has been validated as an important indicator for predicting fall risk [30,31]. Previous studies simply used PEs in EEG as physiological signal analysis but not in TUG; however, we not only discuss the features availability but also the features performance of the decision making.

This work therefore aims to supplement professional assessments of fall risk by developing posture stability assessment methods using sensor technology, entropy features, and statistical analysis. Further, this work aims to understand the availability of the sensor-provided raw data, as well as calculate the availability of PE and WPE in the postural stability, trying to discuss the use of practical medical practice. Additionally, the suitability of utilizing PE to measure gait dynamics is investigated based on the repeatability and sensitivity to changes in walking conditions [32]. Overall, this work aims to provide a clinical, meaningful, and easy-to-use fall-risk diagnosis method that uses sensing technology.

## 2. Materials and Methods

Participants were equipped with a waist-mounted triaxial accelerometer to perform the TUG walking test; the data were compared with the short-form Berg balance scale (SFBBS), used to assess a community-dwelling elderly person’s postural stability. The SFBBS, psychometrically similar (including test reliability, validity, and responsiveness) to the original BBS [33], is widely used to estimate a person’s fall risk by determining their static and dynamic balancing abilities and providing a sound measurement for balance impairment [16]. The sensor data were analyzed through PE and WPE methods.

### 2.1. Subjects

Ninety-one community-dwelling elderly people living in a community in central Taiwan were recruited between April 2014 and May 2015 to perform the SFBBS and TUG test to evaluate their postural stability. The medical professional team included rehabilitation physicians, physiotherapists, and functional therapists. The subjects were all over 65 years of age, had no history of musculoskeletal injuries or central nervous system problems in the prior three months, could walk independently without any help, and completed a written consent form prior to testing. Due to the lack of signal acquisition, data were only collected for 85 subjects, aged 76.12 ± 6.99. Among the subjects, there were 18 men, aged 78.89 ± 5.95, and 67 women, aged 75.37 ± 7.1. It is worth noting that the collection of the elderly was not easy, which rendered achieving a balance of gender restrictions difficult.

### 2.2. Sensors Used

**Figure 1:**
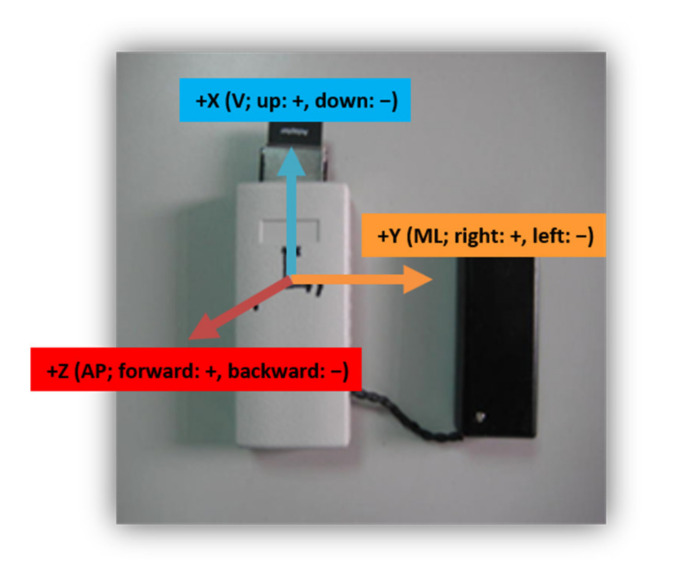
Corresponding axes/directions of the triaxial accelerometer.

During the TUG test, a triaxial accelerometer (RD3152MMA7260Q; Freescale Semiconductor-NXP, Austin, TX, USA; sampling rate: 45 Hz) was placed on the subject’s back at vertebrae L3–L5, as this location is the center of gravity of the human body and is commonly used in fall-risk assessment studies [11]. The X, Y, and Z axes were aligned with the vertical (V; up: +, down: −), mediolateral (ML; right: +, left: −) and anterior−posterior (AP; forward: +, backward: −) directions, respectively, as shown in Figure 1.

### 2.3. Clinical Testing

To reduce the screening work of front-line personnel and improve their objective decision-making skills, measurements were performed on community-dwelling elderly persons using both clinical tests and inertial sensors. Fall-risk analysis methods used included inertial sensor statistical indicators of the accelerator, PE and WPE analysis, multivariable logistic regression, and calculation of the area under the receiver-operating characteristic (ROC) curve (AUC).

**Figure 2:**
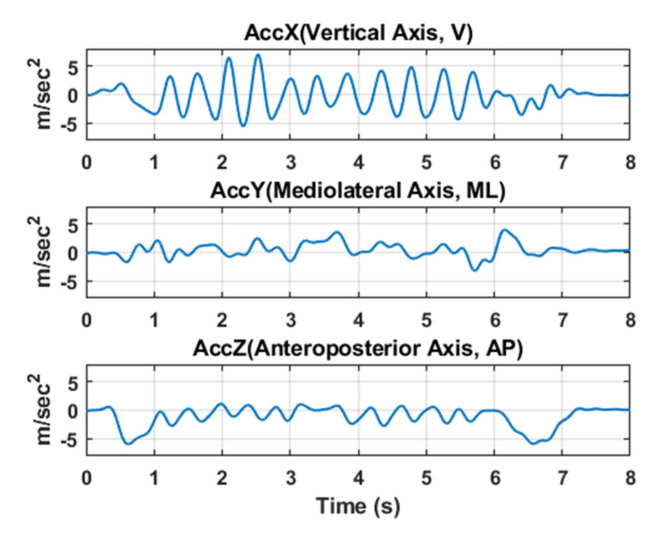
Example data obtained during the timed up and go (TUG) test, filtered through a sixth-order Butterworth and a 3 Hz low-pass filter.

On an average, each subject completed all fall assessment tests within 15−20 min. The SFBBS test, which takes half as long as the BBS, contains seven activities [34]: (1) stretching arms forward; (2) standing with eyes closed; (3) standing with one foot in front; (4) turning and looking backward; (5) picking an object up from the floor; (6) standing on one foot; and (7) standing up from a seated position. During testing, clinicians scored each item for a maximum of four points (for a maximum total score of 28). Prior studies employing SFBBS have classified subjects receiving scores < 23 as having an impaired balance [16,35]. Each subject then performed the TUG test. The observer marked the start time and end time, as well as the time to reach the standing position, reach the three-meter mark, turn around, reach the chair, and then return to the sitting position, while the raw data were collected from the triaxial acceleration sensor; an example of this raw data is shown in Figure 2. The signals of the fall risk data were filtered using a sixth-order Butterworth filter and a low-pass filter with frequency of 3 Hz, as suggested by [25,36].

### 2.4. Data Analysis

Computation of the PE and WPE, *t*-testing of significant features, regression, and statistical analysis were carried out using SPSS and MATLAB^®^. The complexity of the TUG test for each subject was obtained by analyzing the PE, WPE, and statistical features (SFs), including the mean, standard deviation, maximum, minimum, and zero-crossing rate (ZCR), accounting for the three axes; there was a total of 21 features studied. The selection of the above features depends on the reference referred to as the window size impact in human activity recognition; these are some of the features most widely used in activity recognition [37,38,39,40,41]. Among them, the zero-crossing rate is the number of times the signal passes the average value.

**Figure 3:**
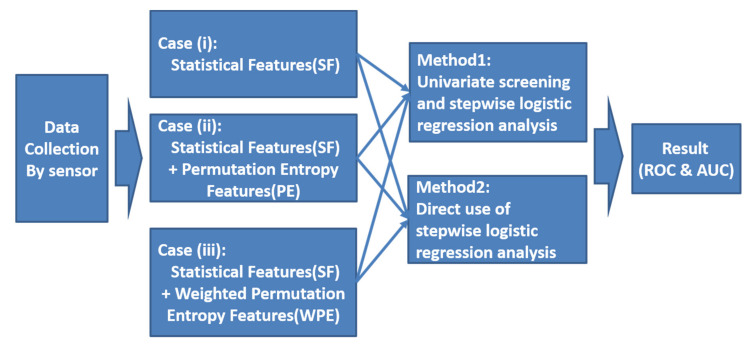
Flow chart of the data analysis of statistical features (SFs), permutation entropy (PE), and weighted-permutation entropy (WPE).

The rest of the article is organized considering that the statistical analysis includes univariate screening, multivariable logistic regression, ROC, and AUC, which were used to assess not only the fall-risk prediction tool but also the performance metrics of the fall-risk regression model. Thus, in the present study, we proposed three cases with SFs, PE, and WPE to compare the selected features in Methods, whereas the ROC curve and AUC were calculated to estimate the postural stability and entropy. This novel approach was selected to characterize the differences between fall-risk and non-fall-risk subjects, confirm the fall-risk prediction tool, and compare the selected features and their impact on decision making. A flow chart of the data analysis of SF, PE, and WPE in this study is provided in Figure 3.

#### 2.4.1. Entropy Analysis

PE and WPE analyses were performed; the methodology is presented in the following subsections.

##### PE

The PE estimates the complexity of a time series based on the relative frequency of the sequence patterns for a time series *x* and data length *N*, as $x=\left\{x_{0},x_{1},\ldots x_{N-1}\right\}$. The PE is computed in three major steps, detailed below [26].

(1) Partitioning the state space:

Firstly, the one-dimensional time series is partitioned into a matrix of overlapping vectors. This partitioning uses two hyperparameters: embedded dimension *m* and time delay τ, where τ=1 to avoid missing any patterns. These hyperparameters are used to extract time series $x_{j}^{m}=\left\{x_{j},x_{j+1},\ldots x_{j+m-1}\right\}$ and form *N −* (*m* − 1) possible subsequences, starting at index *j*, where $0\leq j\leq \left(N-m+1\right).$

(2) Finding the ordinal patterns:

After partitioning the one-dimensional time series, the subsequences of vector *x* are mapped into unique permutations and sorted in ascending order that captures the ordinal rankings of the data, which can then be used to distinguish corresponding ordinal patterns. There are *m*! different possible ordinal patterns of length *m*, termed $\pi_{i}=\left\{\pi_{0},\pi_{1},\ldots \pi_{m!-1}\right\}$, beginning at index *i* with 0≤i≤m!−1.

(3) Calculating the relative frequencies and entropy:

For all possible *m*! permutations, each probability $p(\pi_{i})$ was estimated by calculating the relative frequency of each ordinal pattern and then the PE, shown in the equation below. Here, a lower PE corresponds to a more regular time series [27], as shown in Equation (1):

(1) $$\mathrm{PE}=-\sum_{i=0}^{m!-1}p\left(\pi_{i}\right)\log_{2}\left(p\left(\pi_{i}\right)\right),\;\mathrm{if}\;p\left(\pi_{i}\right)>0,$$

$$\mathrm{normalized}\;\mathrm{PE}=\mathrm{PE}/\log_{2}\left(m!\right).$$

In a previous study, the embedding dimension (*m*) selection of the signal ranged between 3 and 6, indicating that the appropriate embedding dimension (*m*) is related to the signal and its sampling frequency, while it was shown that short permutations cannot capture the entire dynamics [27,42]. Therefore, we tried to select *m* = 6 to obtain richer information. Moreover, we used adaptive resampling with interpolation to overcome the data length issue. In signal processing, oversampling is the process of sampling a signal at a sampling frequency that is significantly higher than the Nyquist rate. In general, the Nyquist frequency is half of the sampling rate of a discrete signal processing system. Thus, herein, the sensor sampling rate was 45 Hz and the Nyquist frequency was 22.5 Hz, implying that the data will not be oversampled at the motive signal behind filtering (3 Hz). Furthermore, to avoid the setting of m, we tried to calculate AUC when using *m* = 3 as an example in the “univariate screening and stepwise logistic regression analysis” method (Section 3.1). When *m* = 3 was selected, the obtained AUC in case (ii) was lower than when *m* = 6 was selected; however, case (iii) had almost the same AUC at both values of m, therefore, we selected *m* = 6. The PE was normalized to a value between 0 and 1 (0 ≤ PE ≤ 1), which facilitated the comparison between the permutation entropies in the present study [28].

##### WPE

The WPE, which was proposed by Fadlallah et al. [29], accounts for the variability of the amplitude information by applying a correcting factor or weight to the relative frequencies that takes into account both the sample variability and order. Thus, the weight (*w*) depends on the variation of the subsequences of vector *x* for the embedded dimension *m*, as shown in Equation (2):

(2) $$w_{j}=\frac{1}{m}\sum_{k=1}^{m}{\left[x_{j+\left(k-1\right)}-{\bar{X}}_{j}^{m}\;\right]}^{2},$$

where

$${\bar{X}}_{j}^{m}=\frac{1}{m}\overset{m}{\underset{k=1}{\sum }}x_{j+\left(k+1\right)}$$

denotes the arithmetic mean of ${\bar{X}}_{j}^{m}$ beginning at index *j*, where 0≤j≤N−m+1.

Thus, the modified $p(\pi_{i})$ can be considered as the proportion of the variance accounted for by each ordinal pattern, denoted as $p_{w}(\pi_{i})$ [29], as expressed in Equation (3):

(3) $$\mathrm{WPE}=-\sum_{i=0}^{m!-1}p_{w}(\pi_{i})\log_{2}\left(p_{w}(\pi_{i})\right),\;\mathrm{if}\;p_{w}(\pi_{i})>0,$$

$$\mathrm{normalized}\;\mathrm{WPE}=\mathrm{WPE}/\log_{2}\left(m!\right),$$

The parameter, *m*, and data length (N) of WPE are the same as those of PE.

##### Adaptive Resampling Procedure in PE/WPE

**Figure 4:**
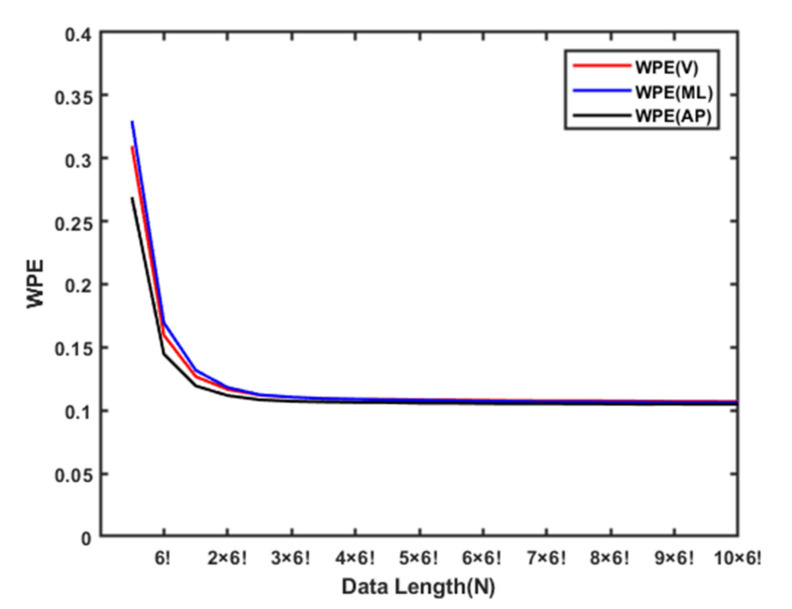
An example of WPE vs. data length (N) with the interpolation of adaptive resampling.

The data length must be considered when performing the PE/WPE analysis; typically, the time series of the length satisfies N > 5*m*!. For example, when *m* = 6 is selected, the data length should be more than 3600 points. It is reasonable to assume that with a high sampling rate, a detailed variation of PE/WPE can be achieved. Figure 4 shows an example of the variation of WPE with the number of data points. It is observed that the WPE decreases with the number of data points; however, data points greater than 5 × 6! have almost constant WPE. During the short sequences of the TUG tests based on the resampling process, we could show the detailed variation in the PE/WPE analysis and extract the local microstructure feature [43], which would help evaluate the fall risk of the elderly in the community.

### 2.5. Statistical Analysis

In our present study, we used the candidate predictors (variables) that were carefully selected based on prior knowledge (*p* < 0.05) and were used as inputs to multivariate logistic regression models to determine which features could be used to classify subjects as a fall risk; the results were compared with SFBBS criterion. The dependent variable in the multivariable logistic regression analysis was the fall-risk classification. We adapted the stepwise logistic regression with backward elimination using a *p*-value criterion of 0.157, which is suitable for prognostic models [44]. The ROC curve, well developed in the field of medicine [45], was also created to further explore the ability of clinical measures and complex index values to predict fall risk. Here, AUC = 0.5 indicates no discrimination, AUC = 1.0 indicates perfect discrimination, and 0.7 ≤ AUC ≤ 0.9 indicates an acceptable level of discrimination. The functional outcomes of the clinical test were compared using a student *t*-test, where the statistical analysis was considered significant if p≤0.05.

## 3. Results

The discussion and analysis address the internal SFs, PE, WPE, and stepwise logistic regression analyses in three main parts. After first classifying each subject as a fall risk or a non-fall risk using the SFBBS criterion, univariate screening and multivariate analyses were performed, as detailed in Section 3.1, using *t*-test analysis to verify the categorization of fall risk, for example, medical experts’ decision as the same as selecting the significant features. Stepwise logistic regression was then performed. Next, stepwise logistic regression analysis was the automatic variable selection with *p* < 0.05; this is discussed in Section 3.2. Finally, these two methodologies are compared in Section 3.3. Additionally, the AUC of the logistic regression results was calculated to understand the decisive features that are actually similar to the results of the clinical tests as predictions for decision making.

### 3.1. Univariate Screening and Stepwise Logistic Regression Analysis

In accordance with medical experts, the subjects were considered as presenting a risk of fall if their total SFBBS score was <23. Of the 85 subjects, 19 were classified as a fall risk according to SFBSS with an age of 78.37 ± 7.54; 66 were classified as non-fall risk, with an age of 75.47 ± 6.74.

**Figure 5:**
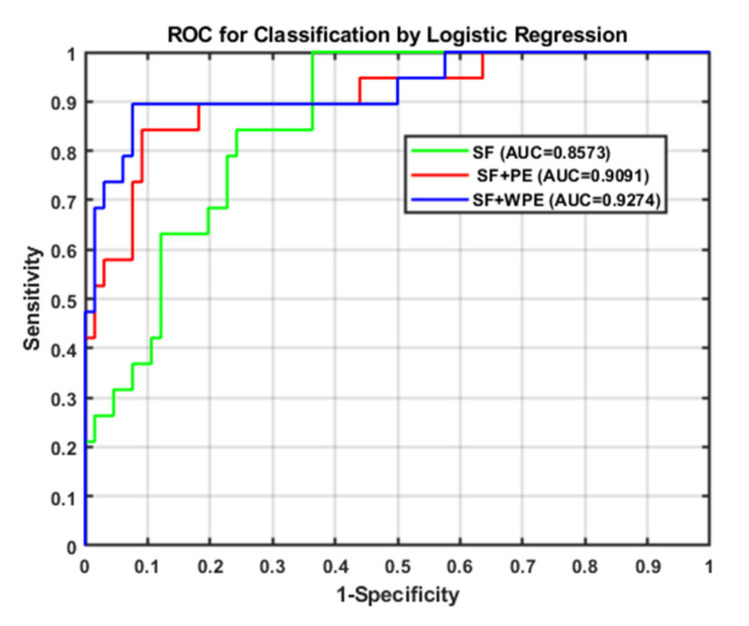
Resulting the receiver-operating characteristic (ROC) curve and area under the curve (AUC) after univariate screening and subsequent stepwise analysis.

The results and discussion are divided into three cases: (i) SFs, (ii) SFs and PE, and (iii) SFs and WPE. Stepwise logistic regression was then performed for each case and calculated AUC for comparison. The results of the *t*-tests for each of the 21 features are detailed in Table 1. After all eigenvalues were tested by *t*-test (*p* ≤ 0.05), they were included in the stepwise regression, summarized in Table 2 and detailed in Table 3. Table 3 shows each case’s selected significant features when performing the omnibus test of logistic regression model coefficients, which is equivalent to the ANOVA-F test in linear regression. All of the β coefficients were zero, indicating that the model is significant and has predictive ability. When checking the odds ratio (Δ odds, expressed as EXP(B) in SPSS), variables with *p* < 0.157—as suggested in a previous work where the stepwise logistic regression with backward elimination was implemented—were used, which is suitable for a prognostic model [44], indicating a direct relationship with the risk of falling. In case (i), F1 and F9 were both significant, in case (ii), F9 and F18 were significant, and in case (iii), F1, F20, and F21 were significant. The ROC curve and AUC were then calculated as an indicator to judge the overall predictive ability of the model; they are shown in Figure 5, where the calculated AUC of cases (i), (ii), and (iii), are 0.8573, 0.9091, and 0.9274, respectively. Thus, case (iii) (i.e., SFs and WPE) offered the best performance, indicating that the larger the AUC value of the classifier, the higher the accuracy: AUC = 0.5 indicates no discrimination, 0.7 ≤ AUC ≤ 0.8 indicates acceptable discrimination, 0.8 ≤ AUC ≤ 0.9 indicates excellent discrimination, and 0.9 ≤ AUC ≤ 1.0 indicates outstanding discrimination.

### 3.2. Direct Use of Stepwise Logistic Regression Analysis

Next, stepwise logistic regression was performed directly (i.e., the features were not pre-selected) to determine the predictive factors. The resulting features selected by the regression are shown in Table 4.

**Figure 6:**
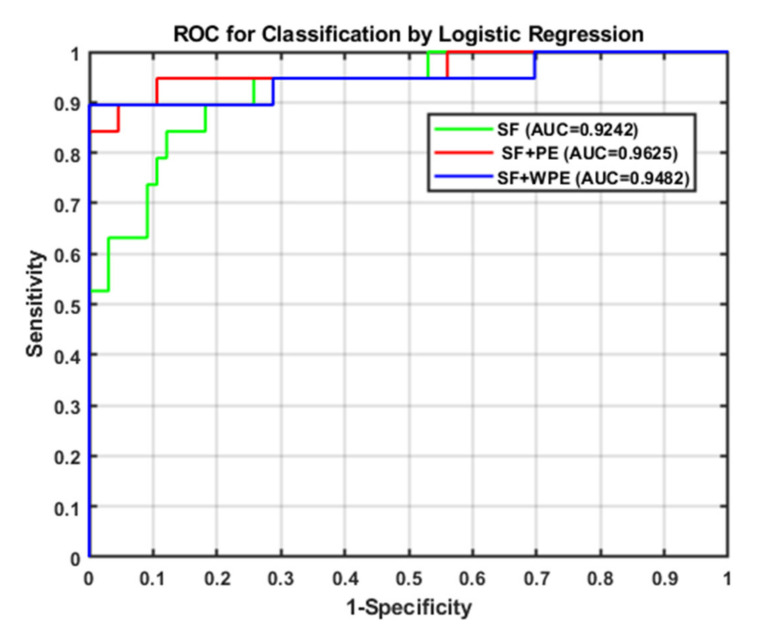
Resulting ROC curve and AUC after direct stepwise analysis.

Similarly, the ROC curve and AUC were calculated to classify the logistic regression; the resulting AUCs for cases (i)–(iii) were 0.924, 0.963, and 0.948, respectively, as shown in Figure 6. Thus, case ii (i.e., SFs and PE) offered the best performance, indicating that the larger the AUC value of the classifier, the higher the accuracy.

### 3.3. Comparison between the Subsequent and Direct Logistic Regression Methods

In this section, the results in Section 3.1 and Section 3.2 (i.e., using univariate screening and auto selection) are compared. At first, we can see that the cases result seems different; however, the entropy makes more sense for predict, indicating that the models that included PE or WPE features are more accurate than the model that included only standard SFs. When using univariate screening and subsequent regression, case iii exhibited high accuracy; the selected features were F1, F9, F15, F20, and F21. When using direct regression, case ii demonstrated the best predictive abilities; the selected features were F1, F2, F3, F4, F6, F12, F14, and F18. Only F1 was selected in both methods, and the anterior−posterior (AP; forward: +, backward: −) axis is the most significant to be selected. The results from the confusion matrix (Table 5) indicated that the inclusion of PE or WPE improved the specificity; however, 100% specificity was not achieved. Therefore, this more precise model would decrease the rate of false positives, leading to a lower sensitivity. Therefore, some patients with risk of falls could not be identified.

## 4. Discussion

Fall risk has commonly been determined using inertial sensors as assessment tools to calculate physiological values, such as gait step and gait speed [9,12,19,20,21]; however, these values are affected by the acceleration and displacements of different axes such as gauges and gyroscopes. Additionally, they are less calculated by the sensor itself. This work therefore aimed to clarify the sensor values using PE and WPE during feature selection to predict fall risk, rather than focusing on stopwatch feedback.

As regression analysis was used in different ways to explore the prediction results, the variable selection is a significant area of interest [46]. Predictive regression models have been used to explore dependent variables (sensor features). Compared with previous explorations of the importance of the independent variable, there will be different statistical models and thinking. Taking this research as an example to discuss dependent variables, all possible feature extractions are included in the way, and feature selection methods used by statistics of the *t*-test are a way to determine the feature sets. Thus, we discuss the model “content” and obtain the best model performance and results. Howcroft et al. [11] reviewed three main methods and classified how often the methods are used to examine subjects’ fall risk: retrospective fall history (30%), prospective fall occurrence (15%), and scores on clinical assessments (32.5%). In particular, fall history and clinical assessments are more commonly used; however, only clinical assessments can be analyzed, and based on the results of this study, the use of sequential univariate screening and logistic regression adopted model building and bivariate analysis, and selected all variables with *p* < 0.05 into the multivariate analysis, which is very common (and even widely accepted) in current journals. The study of model establishment methods [24] showed that joining WPE can achieve better performance. Prior researchers have indicated that “univariate screening and multivariate analyses” is prone to false positives (i.e., a false indication of correlation); here, false positives were avoided using the SFBBS first criterion. The results discussed in Section 3.1 “Univariate Screening and Stepwise Logistic Regression Analysis ” seems to be in line with prior researchers’ [26] findings that WPE can overcome the shortcomings of PE and is more effective. However, looking at the results of “Direct Use of Stepwise Logistic Regression Analysis for Feature Selection”, among them, the eigenvalues that are not significant (*p* > 0.05) are also in the model. Moreover, as demonstrated in the result of Section 3.2, in terms of statistical interpretation, the eigenvalues are in the hyperplane formed by multi-dimensional projections, which has the best performance. Therefore, in data science, the results discussed in Section 3.2 are considered a reasonable mathematical model as a predictive result. However, in the field of biomedicine, such as the study of risk of falls, it is difficult to explain why non-significance is an important feature, because medical experts are unconcerned with the non-significant features, although some studies state that it “may be” a potential factor. Regardless, if there is a criterion that can be used as the first step of bivariate analysis, it may help prevent false positives.

Based on the prediction results of the two, starting from different prediction models led to inconsistent results; however, adding entropy as a feature value led to improved overall predictive abilities than simple SFs. PE and WPE can thus be used during real-time analysis of the intrinsic sensor to implement the real-time analysis of practical services, such as community services. In addition, from the result of feature selection, F1 (mean_V) has been selected in two prediction approaches. The features of the AP axis offer the most predictive abilities in the prediction model. Thus, the sensor can detect the shaking of AP axis that is difficult to recognize with the naked eye, and it looks more like maintaining proprioception.

Researchers have demonstrated that multifactorial analysis is most effective at predicting older adults’ fall risk. [47]. This work therefore aimed to discuss which sensor feature readings can most accurately be used to predict fall risk. Employing intrinsic sensors to explore the availability of posture control has been widely studied [48], hoping to extend the discussion on how to use significant features to effectively predict the risk of falls. It is worth mentioning that the collection of data from the elderly is very difficult; thus, it is not easy to achieve a balance of gender restrictions. Therefore, the discussion on gender differences is more limited. Overall, in terms of decision making, we must first understand the purpose, because different goals will lead to different forecasting methods, and the results will certainly be different. This research attempts to explain the results of different positions.

## 5. Conclusions

In this work, WPE and PE were applied to inertial sensor signals to predict the fall risk of elderly people. Stepwise logistic regression analysis was applied to present the inertial data on a fall-risk scale with different perspectives to allow medical practitioners to screen for high-risk patients. The logistic regression was also used to automatically select feature values and clinical judgment methods to explore the differences in decision making. The results indicated that directly applying stepwise logistic regression to obtain PE and SF provided the best AUC value and avoided false positives. Additionally, employing WPE and SF obtained by using the clinical test (SFBBS) as golden rule of predictive value can be more similar to the decision-making reference of clinical experts. Therefore, the proposed methodology can provide decision-makers with a more accurate manner to classify fall risks in elderly people.

## Figures and Tables

**Table 1:** *T*-test results verifying the categorization of fall risk by the short-form Berg balance scale (SFBBS) criterion.

		Fall Risk (*n* = 19)	Non-Fall Risk (*n* = 66)	*p* Value
**Statistical Feature**		**Average ± Standard Deviations**		
F1	Mean_V	1.0378 ± 0.2909	1.4961 ± 0.4715	0.000 **
F2	Mean_ML	1.0816 ± 0.2842	1.0539 ± 0.2332	0.701
F3	Mean_AP	2.0050 ± 0.6843	1.6563 ± 0.5206	0.051
F4	Std_V	1.3346 ± 0.3932	1.8947 ± 0.5791	0.000 **
F5	Std_ML	1.2005 ± 0.3085	1.3152 ± 0.2835	0.158
F6	Std_AP	1.7621 ± 0.3512	1.9474 ± 0.3279	0.049 *
F7	Max_V	4.4866 ± 1.6012	5.2472 ± 1.6480	0.080
F8	Max_ML	3.1687 ± 1.0598	3.6300 ± 1.2326	0.117
F9	Max_AP	1.3401 ± 0.8068	2.4738 ± 1.2365	0.000 **
F10	Min_V	−3.6991 ± 1.3737	−4.4409 ± 1.3678	0.047 *
F11	Min_ML	−3.0965 ± 1.0994	−3.5054 ± 1.0356	0.159
F12	Min_AP	−7.5277 ± 1.1730	−6.9939 ± 1.3935	0.104
F13	ZCR_V	0.0984 ± 0.0169	0.0906 ± 0.0123	0.073
F14	ZCR_ML	0.0659 ± 0.0148	0.0847 ± 0.0203	0.000 **
F15	ZCR_AP	0.0528 ± 0.0158	0.0651 ± 0.0137	0.005 **
**PE Feature**		**Average ± Standard Deviations**		
F16	PE_V	0.1108 ± 0.0007	0.1103 ± 0.0006	0.028 *
F17	PE_ML	0.1105 ± 0.0006	0.1107 ± 0.0007	0.291
F18	PE_AP	0.1105 ± 0.0016	0.1093 ± 0.0011	0.006 *
**WPE Feature**		**Average ± Standard Deviations**		
F19	WPE_V	0.1048 ± 0.0007	0.1050 ± 0.0004	0.172
F20	WPE_ML	0.1043 ± 0.0012	0.1049 ± 0.0008	0.043 *
F21	WPE_AP	0.1041 ± 0.0012	0.1029 ± 0.0019	0.002 **

* indicates *p* < 0.05 between two groups. ** indicates *p* < 0.005 between two groups.

**Table 2:** Significant features of univariate screening and multivariate analyses.

Method	Feature Group	Selected Features
Stepwise (used *t*-test (*p* ≤ 0.05)	SFs	F1, F9
	SFs and PE	F1, F4, F9, F14, F18
	SFs and WPE	F1, F9, F15, F20, F21

**Table 3:** Stepwise logistic regression results for each case.

	Omnibus Test	Δ Odds (EXP(B)) and Significance
Case i	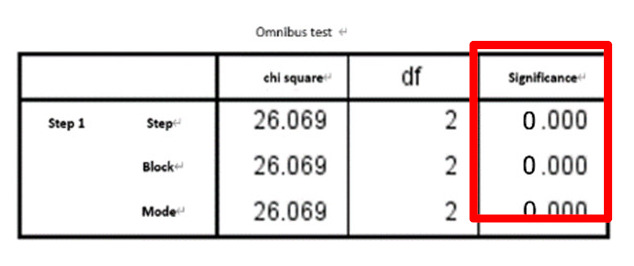	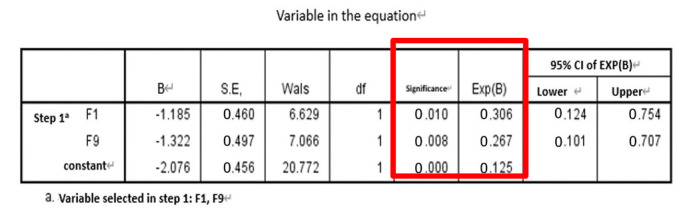
Case ii	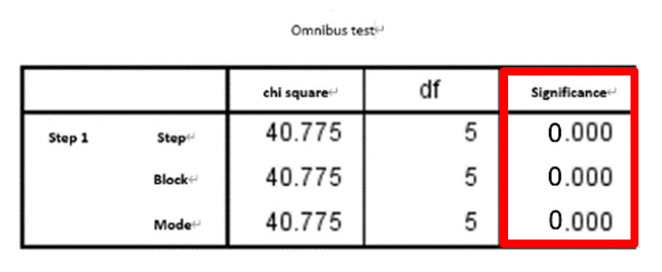	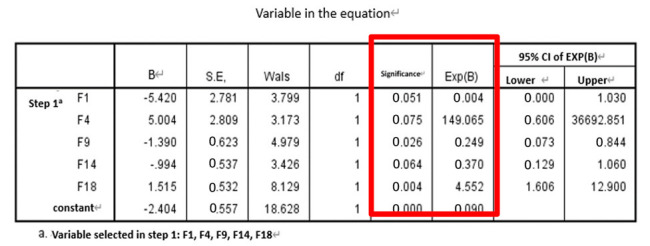
Case iii	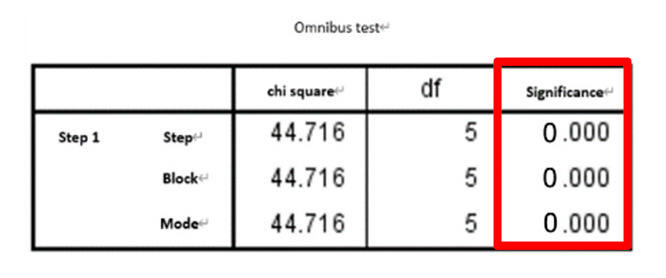	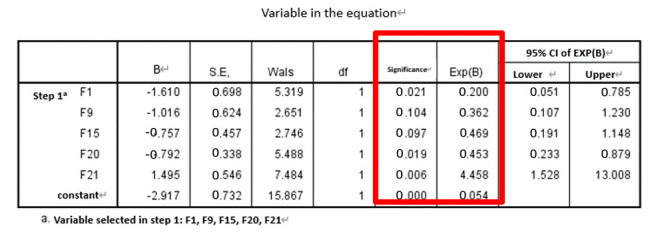

**Table 4:** Significant features selected via direct stepwise logistic regression.

Method	Feature Group	Selected Features
direct stepwise logistic regression	SF	F2, F5, F6, F12, F15
	SF and PE	F1, F2, F3, F4, F6, F12, F14, F18
	SF and WPE	F2, F5, F6, F12, F15, F20, F21

**Table 5:** Confusion matrix of sensitivity, specificity, and accuracy for each case.

	Sensitivity	Specificity	Accuracy
SF	100%	63.6%	71.8%
SF and PE	84.2%	89.4%	88.2%
SP and WPE	89.5%	92.4%	91.8%
